# Investigation on the knowledge-attitude-practice of medical students in controlling emerging infectious diseases: A case study of COVID-19

**DOI:** 10.1371/journal.pone.0338708

**Published:** 2025-12-10

**Authors:** Yizhe Yang, Ruifeng Liang, Yan Luo, Doudou Zhu, Yi Liu, Yuyan Guo, Jiafen Zhang, Qiao Niu

**Affiliations:** 1 Department of Environmental Health, School of Public Health, Shanxi Medical University, Taiyuan, China; 2 MOE Key Laboratory of Coal Environmental Pathogenicity and Prevention, Shanxi Medical University, Taiyuan, China; 3 Research Center for Ecological Public Health and Safety in the Yellow River Basin, Shanxi Medical University, Taiyuan, China; 4 Key Laboratory of Cellular Physiology (Shanxi Medical University), Ministry of Education, Taiyuan, China; 5 Department of Occupational Health, School of Public Health, Shanxi Medical University, Taiyuan, China; Riphah International University, PAKISTAN

## Abstract

**Objective:**

Investigate the Knowledge-Attitude-Practice (KAP) of students from Medical College towards emerging infectious diseases, and assess their impact, can provide a scientific basis and practical guidance for enhancing medico’s prevention and control capabilities.

**Methods:**

A total of 2,395 participants from various grades and majors at Medical University were randomly selected using a stratified cluster sampling method. This cross-sectional study was conducted between April 25 and May 31, 2020, using a self-administered questionnaire developed on the Wenjuanxing platform to assess COVID-19-related knowledge, attitudes, and practices (KAP) among medical students.

**Results:**

A total of 2,245 participants (aged 16–28 years) were included in the study, coming from five medical disciplines: Clinical Medicine, Preventive Medicine, Nursing, Clinical Pharmacy, Health Inspection and Quarantine. The average scores for the COVID-19 epidemiological knowledge and the control measures for the epidemic were 4.92 ± 1.03 and 4.50 ± 0.78, respectively. Among them, the scores of epidemiological knowledge exhibited significant differences in sex, nation, type of dwelling place, major, grade, annual per capita household income, and age. The scores of preventive knowledge significantly differed by sex, major, grade, physical condition, and age. Further, behavioral data indicated that 96.0% of the students thought the pandemic had severely affected their daily life, while >90% maintained consistent mask usage and >80% insisted on health-protective practices. Practice scores finally varied significantly by sex, family structure, and ethnicity.

**Conclusions:**

Altogether, medical students possess certain basic knowledge in controlling emerging infectious diseases, but some still generally suffer from insufficient cognitive depth and anxiety. Colleges can systematically enhance students’ rational cognitive level which include offering specialized courses as well as promoting cutting-edge research achievements, and through standardized operations stabilize their psychological states.

## 1. Introduction

As a significant threat to global health, infectious diseases are exerting substantial morbidity and mortality across diverse people and regions [1,2]. Recent outbreaks of Emerging Infectious Diseases (EIDs) have not only compromised global health security systems but also caused profound disruptions to the operation of the economy and society [3]. The increasingly complex transmission mechanisms of pathogens have a significant impact on public health response capacities [4]. EIDs demonstrate marked unpredictability and abrupt onset characteristics [5], typically manifesting clustered transmission patterns during the initial stage. These pathogens exhibit highly complex transmission dynamics, with rapid dissemination through multiple ways including respiratory droplets, close contact, aerosols, and fecal-oral pathways [6,7] among susceptible populations. This pattern can be confirmed by the early COVID-19 pandemic. The COVID-19 pandemic displayed exponential spread that highlighted both typical epidemiological characteristics and corresponding containment challenges of EIDs [8]. Meanwhile, the outbreak corroborated key EID transmission attributes: high dissemination efficiency, pre-symptomatic dissemination potential, and multimode dissemination – all contributing to significant early-phase control difficulties.

The WHO prioritizes several pathogens with pandemic potential, including COVID-19, Crimean-Congo hemorrhagic fever, Ebola, Marburg virus, Lassa fever, MERS, SARS, Nipah and henipaviral diseases, Rift Valley fever, Zika, and Disease X [9]. Among them, COVID-19 exemplifies zoonotic transmission, and actually, most emerging diseases in the past century originated from animals [10]. The 2014 Ebola, for instance, began with a Guinean boy who contracted the virus from infected bats, leading to familial transmission [11]. Lassa virus typically spreads through exposure to excreta of Mastomys rodents [12], while Nipah virus first emerged during Malaysia’s 1999 outbreak [13]. Genomic evidence implicates bats and pangolins as probable reservoirs for SARS-CoV-2’s progenitor viruses [14]. The absence of targeted therapies contributes to high morbidity and mortality - Ebola’s [11] fatality reaches 25–90%, and Nipah’s [13] 40–75%, probably, making KAP-based prevention a critical global health imperative.

The knowledge, attitude, and practice (KAP) model, a theoretical framework for modifying health-related behaviors [15], constitutes an essential component of optimal infectious disease prevention strategies for prior research on EIDs, when its levels have been evaluated and enhanced. Ebola’s case [16] and MERS’s [17] have demonstrated that. Population-level KAP regarding infectious diseases plays a pivotal role in outbreak prevention, so strengthening community preparedness and offering knowledge timely manner can promote healthier behavior as well as improve overall epidemic response capacity [18].

Schools function as high-density social systems organized for educational purposes, bringing together students, teachers, and other participants within defined spatiotemporal parameters. These concentrated settings serve dual roles: enabling efficient allocation of educational resources while simultaneously acting as high-risk transmission nodes, making them epidemiologically critical during emerging infectious disease (EID) outbreaks [19]. The COVID-19 pandemic impacted nearly every country and region worldwide within a similar timeframe, creating a near-ideal foundation for comparative research while also revealing challenges across multiple societal dimensions. This study focuses on medical students as the research population. Given the congregate nature of the campus environment, implementing scientific preventive measures proves critical for containing respiratory disease transmission like COVID-19 [20]. In this study, the survey was conducted from April 25 to May 31, 2020, during the phase of regularized COVID-19 prevention and control in Chinese universities. During this period, higher education institutions implemented orderly and staggered-return policies to facilitate the safe return of students while prioritizing the resumption of classes for key groups such as graduating seniors. A blended online and offline teaching model was adopted to avoid one-size-fits-all management approaches. Meanwhile, comprehensive measures were enforced to enhance environmental hygiene and material support, including routine disinfection, temporary isolation facilities, and adequate provision of epidemic prevention supplies to safeguard the health of students and staff. Current epidemiological surveillance indicates that while academic operations have resumed, latent transmission risks persist in schools [21]. Enhancing students’ health literacy, particularly KAP regarding infectious diseases, can effectively mitigate infection risks. Notably, as EID transmission mode evolves, university students’ KAP levels directly impact campus public health security that underscoring health education’s foundational role in epidemic prevention systems [22].

The epidemiological complexity and public health hazards posed by EIDs necessitate a preventive-focused, forward-looking containment strategy. Within this strategy, medical students constitute both a potentially vulnerable group in disease transmission and a critical frontline force in future outbreak response. Meanwhile, as healthcare system trainees, their KAP regarding EID prevention, such as COVID-19, directly influences public health emergency preparedness [23]. This cross-sectional study has employed online questionnaires to assess COVID-19-related KAP patterns and determinants among medical students at Shanxi Medical University. The findings are designed to provide evidence-based insights for refining educational interventions in EID prevention and offer scientific references for developing targeted campus containment measures to enhance future healthcare workers’ epidemic response capabilities.

## 2. Methods

### 2.1. Study design and participants

From April 25 to May 31, 2020, a cross-sectional study of medical students across different grades and majors at Shanxi Medical University was conducted. The study employed stratified cluster random sampling, first selecting students from clinical medicine, preventive medicine, clinical pharmacy, nursing, and health inspection and quarantine programs. Researchers then randomly sampled 120–150 students from each academic year, enrolling 2,395 medical students ultimately in the study.

### 2.2. Survey contents

The self-designed questionnaire comprised two sections: sociodemographic characteristics and the KAP questionnaire. The sociodemographic characteristics included sex (male/female); age; major (clinical medicine, preventive medicine, nursing, clinical pharmacy, health inspection and quarantine); grade (freshman, sophomore, junior, senior, and fifth grade); type of dwelling place (municipality/provincial capital, prefecture-level city, countryside, county town); parental occupational type and annual per capita household income (<10000 yuan, 10000–13999 yuan, 14000–16999 yuan, 17000–19999 yuan, and ≥20000 yuan).

A 23-item KAP questionnaire was developed to assess medical students’ knowledge, attitude, and practice regarding COVID-19. The instrument based on the COVID-19 Diagnosis and Treatment Protocol (Tentative Version Six) issued by the National Health Commission of the People’s Republic of China on February 18, 2020, structuring it in three sections: (1) an 11-item knowledge section covering epidemiological concepts and preventive measures, (2) a 2-item attitude section evaluating outbreak perceptions and life impacts, and (3) a 10-item practice section assessing behavioral changes in exercise, disinfection, hand hygiene, and mask usage. The Cronbach’s alpha coefficient of the questionnaire was 0.80, indicating acceptable internal consistency.

### 2.3. Score criterion of the KAP questionnaire

A common scoring method was used for the KAP questionnaire as follows.

Knowledge scores were calculated based on the participants’ answers to the knowledge part, with one point assigned to each correct answer and zero points to each wrong answer. Accordingly, the epidemiological and prevention knowledge scores were calculated. The epidemiological knowledge scores range from zero to six, and the prevention knowledge scores range from zero to five, with higher scores representing a better understanding of COVID-19. The awareness rate of knowledge (%) was computed as follows: (number of people who accurately answered COVID-19 knowledge/total number of respondents) × 100%.

For the attitude assessment, the attitude belief prevalence rates to analyze participants’ COVID-19 perceptions. Each belief’s prevalence percentage is calculated using the formula: (number of respondents endorsing a specific belief/total respondents) × 100%. This metric quantitatively describes the distribution of different COVID-19 attitudes among medical students.

In the practice section, changes in health behavior scores were calculated by assigning different scores to each answer: 2 = increased, 1 = no change, and 0 = decreased. Positive choices were made for each option, except for Question 8, and the total score was calculated, ranging from 0 to 20, with higher scores indicating better health practices against COVID-19.

### 2.4. Survey procedures and data collection

This study used stratified cluster random sampling, first grouping students by major (clinical medicine, preventive medicine, clinical pharmacy, nursing, health inspection and quarantine), then randomly selecting 120–150 students from each grade. The final sample included 2,395 medical students. Researchers created the questionnaire using the Wenjuanxing online survey platform (https://www.wjx.cn) and distributed it through WeChat.

Participants were required to provide truthful responses with quality control measures implemented, including mandatory questions, single-IP submission restrictions, and exclusion of questionnaires with inconsistent/incomplete answers, logical errors, or abnormal response times. Valid data were collected through the backend system. Furthermore, the study design and reporting strictly followed the Enhancing the QUAlity and Transparency Of health Research (EQUATOR) guidelines, with cross-sectional components adhering to the Strengthening the Reporting of Observational Studies in Epidemiology (STROBE) statement, including its checklist.

### 2.5. Statistical analysis

Statistical analyses were performed using the statistical software package SPSS V.26.0 for Windows. The basic survey information of the questionnaire was expressed in percentages, and continuous data were described as the mean (M) ± standard deviation (SD). Categorical variables were displayed either as frequencies or percentages. A chi-square test was performed to compare categorical variables between different groups, while a one-way analysis of variance (ANOVA) was used to assess differences in continuous variables. Statistical significance was set at *P* < 0.05.

### 2.6. Ethical statement

The study was conducted under the Declaration of Helsinki and approved by the Ethics Committee of Shanxi Medical University. Electronic informed consent was obtained from each participant before starting the survey. Participation in the study was voluntary, and respondents’ personal and private information was not collected. Moreover, participants were given the option to withdraw from the study at any time without giving any reason.

## 3. Results

### 3.1. Demographic characteristics of subjects

To ensure the validity of the online survey, we applied the following exclusion criteria: inconsistent answers, incomplete answers, and obvious logic errors in responses. Consequently, 150 invalid questionnaires that did not meet the requirements were excluded, leaving a total of 2,245 valid questionnaires (effective recovery rate = 93.7%).

Among respondents, 96.2% were of Han nationality; the average age was 21 ± 1.73 years, with a range from 16 to 28 years. Notably, 1737 were female respondents (77.4%). By major, 805 (35.9%) were in clinical medicine, 435 (19.4%) were in preventive medicine, 667 (29.6%) were in nursing, 132 (5.9%) were in clinical pharmacy, and 206 (9.2%) were in health inspection and quarantine. The cohort included 562 (25.0%) freshmen, 378 (16.8%) sophomores, 428 (19.1%) juniors, 778 (34.7%) seniors, and 99 (4.4%) fifth-graders. More than half of the participants (1,294; 57.6%) were from the countryside (Table 2).

### 3.2. Knowledge of medical students about COVID-19

The average score of comprehensive COVID-19 knowledge was 9.42 ± 1.47 points (range: 1–11 points), and the awareness rate of comprehensive knowledge was 91.2%. The overall average epidemiology knowledge score was 4.92 ± 1.03 points (range: 0–6 points). The average prevention knowledge score was 4.50 ± 0.78 points (range: 0–5 points). The awareness rates of the six epidemiology knowledge questions of “Novel coronavirus officially named,” “Source of COVID-19 infection,” “Transmission route of COVID-19,” “Population susceptible to COVID-19,” “Eclipse period of COVID-19,” and “Isolation period of COVID-19 close contacts” were 71.2%, 96.4%, 91.4%, 67.9%, 70.1%, and 95.0%, respectively. Among the questions for the knowledge related to epidemic prevention measures, the awareness rates of “Choosing the right mask,” “Properly handling the discarded mask,” “Precautions for coughing and sneezing,” “Proper measures to prevent COVID-19,” and “Eliminating the condition of novel coronavirus” were 97.5%, 87.5%, 78.4%, 95.3%, and 91.4%, respectively (Table 1).

**Table 1:** Epidemiology and prevention knowledge scores of COVID-19 among medical students.

Variable categories		Number of correct answers/Total	Percent (%)	Score (x̅ ± s) (point)	Score (x̅ ± s) (point)
**Knowledge of Epidemiology**	Novel coronavirus is officially named	1,599/2,245	71.2	4.92 ± 1.03	9.42 ± 1.47
	Source of COVID-19 infection	2,165/2,245	96.4		
	Transmission of COVID-19	2,051/2,245	91.4		
	People susceptible to COVID-19	1,524/2,245	67.9		
	Eclipse period	1,575/2,245	70.1		
	Isolation period for close contacts of COVID-19	2,133/2,245	95.0		
**Knowledge of Preventive Measures**	Choosing the right mask	2,189/2,245	97.5	4.50 ± 0.78	
	Disposal of discarded masks	1,965/2,245	87.5		
	Precautions for coughing and sneezing	1,759/2,245	78.4		
	Proper prevention measures for COVID-19	2,140/2,245	95.3		
	Eliminating condition of novel coronavirus	2,053/2,245	91.4		

**Table 2:** Comparison of COVID-19 knowledge scores among medical students.

Variable categories		Number of respondents (%)	Knowledge scores of epidemiology			Knowledge scores of preventive measures		
			x̅ ± s (point)	t	P	x̅ ± s (point)	t	P
**Sex**	Male	508 (22.6)	4.79 ± 1.49	2.877	0.004	4.39 ± 0.88	3.422	<0.001
	Female	1,737 (77.4)	4.96 ± 1.00			4.53 ± 0.74		
**Nation**	Han	2,157 (96.1)	4.93 ± 1.03	2.74	0.006	4.51 ± 0.77	1.418	0.160
	Non-Han	88 (3.9)	4.63 ± 1.15			4.36 ± 0.94		
**Type of dwelling place**	Municipality/Provincial capital	245 (10.9)	5.11 ± 1.10	3.614	0.014	4.49 ± 0.84	1.813	0.145
	Prefecture level city	638 (28.4)	4.94 ± 0.99			4.55 ± 0.74		
	Countryside	1,294 (57.6)	4.88 ± 1.05			4.49 ± 0.77		
	County town	68 (3.1)	4.90 ± 1.02			4.32 ± 0.92		
**Major**	Clinical medicine	805 (35.9)	4.87 ± 1.04	5.499	<0.001	4.50 ± 0.80	10.544	<0.001
	Preventive medicine	435 (19.4)	4.81 ± 1.00			4.43 ± 0.75		
	Clinical pharmacy	132 (5.9)	4.95 ± 1.02			4.48 ± 0.87		
	Nursing	667 (29.6)	5.07 ± 1.04			4.62 ± 0.71		
	Health inspection and quarantine	206 (9.2)	4.85 ± 0.99			4.25 ± 0.82		
**Grade**	Freshman	562 (25.0)	4.63 ± 1.04	20.150	<0.001	4.38 ± 0.80	9.134	<0.001
	Sophomore	378 (16.8)	4.89 ± 1.02			4.46 ± 0.84		
	Junior	428 (19.1)	4.92 ± 1.04			4.50 ± 0.78		
	Senior	778 (34.7)	5.12 ± 0.98			4.62 ± 0.70		
	Fifth grade	99 (4.4)	5.15 ± 1.06			4.43 ± 0.83		
**Physical condition**	Healthy	1,774 (79.0)	4.91 ± 1.04	0.785	0.502	4.49 ± 0.78	3.143	0.039
	Fine	389 (17.3)	4.97 ± 1.01			4.59 ± 0.69		
	Commonly	77 (3.4)	4.79 ± 1.00			4.25 ± 1.05		
	Poor	5 (0.3)	4.80 ± 0.84			4.20 ± 0.84		
**Annual per capita household income (yuan)**	<10000	1,188 (52.9)	4.91 ± 1.02	3.531	0.007	4.51 ± 0.77	0.504	0.733
	10000–13999	583 (26.0)	4.93 ± 1.09			4.47 ± 0.79		
	14000–16999	154 (6.9)	4.75 ± 1.05			4.53 ± 0.71		
	17000–19999	105 (4.7)	4.82 ± 0.99			4.50 ± 0.72		
	≥20000	215 (9.5)	5.13 ± 0.96			4.54 ± 0.81		
**Age (year)**	≤19	515 (22.9)	4.69 ± 1.04	17.524	<0.001	4.41 ± 0.81	6.333	0.004
	20–24	1,713 (76.3)	4.99 ± 1.02			4.53 ± 0.76		
	≥25	17 (0.7)	4.71 ± 1.16			4.06 ± 0.97		

Female students had significantly higher scores on epidemiology and preventive measures than male students (*t* = 2.877, *P* = 0.004; and *t* = 3.422, *P* = 0.001, respectively). The average epidemiology score was significantly higher in Han students than in non-Han students (*t* = 2.74, *P* = 0.006). The epidemiology knowledge scores significantly differed among students with different residence types, majors, grades, age groups, and categories of annual per capita household income (*t* = 3.614, *t* = 5.499, *t* = 17.524, and *t* = 3.531, respectively; *P* < 0.05). The knowledge of preventive measures scores significantly differed across sex, major, grade, age group, and physical conditions (*t* = 3.422, *t* = 10.544, *t* = 9.134, *t* = 6.333, and *t* = 3.143, respec*t*ively; *P* < 0.05). However, these differences were not significant in terms of nation, type of dwelling place, and annual per capita household income (Table 2).

### 3.3. Attitudes of medical students toward COVID-19

A total of 731 participants (32.6%) had negative thoughts when they heard about the local outbreak of COVID-19. Those who felt “afraid” and “nervous” were 10.0% (225) and 22.6% (506), respectively. Next, 2,153 respondents (96.0%) believed that COVID-19 had affected their daily life, with 22.9% (514), 57.0% (1,279), and 16.1% (360) respondents saying that COVID-19 had been “Very serious,” “More serious,” and “Serious,” respectively. The proportions of “feeling fear” and “feeling nervous” in female students were higher than those of male students (*χ*^*2*^ = 45.703 and *χ*^*2*^ = 15.021, respectively; *P* < 0.001). Medical students with different types of dwelling places, grades, and physical conditions showed statistically significant differences in “How they felt when they heard about a local outbreak of COVID-19 somewhere” (*χ*^*2*^ = 28.750, *χ*^*2*^ = 26.804, and *χ*^*2*^ = 66.777, respectively; *P* < 0.05). The belief rate of female students that COVID-19 seriously affected their daily lives was higher than that of male students (*χ*^*2*^ = 15.021, *P* < 0.001). Medical students with different majors, grades, physical conditions, and ages showed statistically significant differences in “How much do you think the COVID-19 outbreak has affected your daily life?” (*χ*^*2*^ = 19.481, *χ*^*2*^ = 27.053, *χ*^*2*^ = 31.949, and *χ*^*2*^ = 23.091, respectively; *P* < 0.05) (Table 3).

**Table 3:** Comparison of attitude scores toward COVID-19 by demographics.

Variable categories	Feeling after hearing about a local outbreak of COVID-19				Severity of the epidemic on a daily basis			
	Feel fear	Feel nervous	Keep calm	Not care	Very serious	More serious	Serious	Nothing serious
**Sex**								
**Male**	34 (1.5%)	116 (5.2%)	319 (14.2%)	39 (1.7%)	144 (6.4%)	271 (12.1%)	67 (3.0%)	26 (1.1%)
**Female**	191 (8.5%)	390 (17.4%)	1,121 (49.9%)	35 (1.6%)	370 (16.5%)	1,008 (44.9%)	293 (13.1%)	66 (2.9%)
**χ** ^**2**^	45.703				15.021			
**P**	<0.001				<0.001			
**Type of dwelling place**								
**Municipality/Provincial capital**	17 (0.8%)	58 (2.6%)	154 (6.9%)	16 (0.7%)	58 (2.6%)	134 (6.0%)	39 (1.7%)	14 (0.6%)
**Prefecture level city**	59 (2.6%)	163 (7.2%)	391 (17.4%)	25 (1.1%)	134 (6.0%)	373 (16.6%)	107 (4.7%)	24 (1.1%)
**Countryside**	145 (6.5%)	276 (12.3%)	840 (37.4%)	33 (1.5%)	307 (13.7%)	729 (32.5%)	207 (9.2%)	51 (2.3%)
**County town**	4 (0.2%)	9 (0.4%)	55 (2.4%)	0 (0.0%)	15 (0.7%)	43 (1.9%)	7 (0.3%)	3 (0.1%)
**χ** ^**2**^	28.750				5.953			
**P**	0.001				0.745			
**Major**								
**Clinical medicine**	98 (4.4%)	191 (8.5%)	494 (22.0%)	22 (1.0%)	185 (8.3%)	462 (20.6%)	121 (5.4%)	37 (1.6%)
**Preventive medicine**	34 (1.5%)	87 (3.9%)	301 (13.4%)	13 (0.6%)	88 (3.9%)	252 (11.2%)	78 (3.5%)	18 (0.8%)
**Nursing**	63 (2.8%)	141 (6.3%)	433 (19.2%)	30 (1.3%)	150 (6.7%)	385 (17.1%)	104 (4.6%)	28 (1.2%)
**Clinical pharmacy**	16 (0.7%)	32 (1.4%)	80 (3.6%)	4 (0.2%)	47 (2.1%)	65 (2.9%)	16 (0.7%)	4 (0.2%)
**Health inspection and quarantine**	14 (0.6%)	55 (2.5%)	132 (5.9%)	5 (0.2%)	44 (2.0%)	116 (5.2%)	41 (1.8%)	5 (0.2%)
**χ** ^**2**^	20.192				19.481			
**P**	0.064				<0.001			
**Grade**								
**Freshman**	58 (2.6%)	123 (5.5%)	367 (16.3%)	14 (0.6%)	100 (4.5%)	344 (15.3%)	94 (4.2%)	24 (1.1%)
**Sophomore**	27 (1.2%)	101 (4.5%)	238 (10.6%)	12 (0.5%)	84 (3.7%)	203 (9.0%)	67 (3.0%)	24 (1.1%)
**Junior**	52 (2.3%)	82 (3.7%)	287 (12.8%)	7 (0.3%)	103 (4.6%)	245 (10.9%)	70 (3.2%)	10 (0.4%)
**Senior**	82 (3.7%)	182 (8.1%)	476 (21.2%)	38 (1.7%)	202 (9.0%)	436 (19.4%)	108 (4.8%)	32 (1.4%)
**Fifth grade**	6 (0.3%)	18 (0.8%)	72 (3.2%)	3 (0.1%)	25 (1.1%)	51 (2.3%)	21 (0.9%)	2 (0.1%)
**χ** ^**2**^	26.804				27.053			
**P**	0.008				0.008			
**Physical condition**								
**Healthy**	168 (7.5%)	405 (18.0%)	1,143 (50.9%)	58 (2.6%)	419 (18.7%)	987 (43.9%)	288 (12.8%)	80 (3.6%)
**Fine**	41 (1.8%)	85 (3.8%)	255 (11.3%)	8 (0.4%)	65 (2.9%)	255 (11.3%)	58 (2.6%)	11 (0.5%)
**Common**	16 (0.7%)	16 (0.7%)	40 (1.8%)	5 (0.2%)	29 (1.3%)	34 (1.5%)	14 (0.6%)	0 (0.0%)
**Poor**	0 (0.0%)	0 (0.0%)	2 (0.1%)	3 (0.2%)	1 (0.05%)	3 (0.2%)	0 (0.0%)	1 (0.05%)
**χ** ^**2**^	66.777				31.949			
**P**	<0.001				<0.001			
**Age**								
**≤19**	58 (2.5%)	112 (5.0%)	337 (15.0%)	8 (0.4%)	89 (4.0%)	313 (13.9%)	82 (3.7%)	31 (1.3%)
**20–24**	164 (7.4%)	391 (17.4%)	1,092 (48.7%)	66 (2.9%)	419 (18.7%)	961 (42.8%)	273 (12.2%)	60 (2.7%)
**≥25**	3 (0.1%)	3 (0.1%)	11 (0.5%)	0 (0.0%)	6 (0.3%)	5 (0.2%)	5 (0.2%)	1 (0.0%)
**χ** ^**2**^	9.728				23.091			
**P**	0.111				<0.001			

### 3.4. Medical students’ practice toward COVID-19

As shown in Table 4, the vast majority of the participants disinfected the residence (1,641, 73.1%) and wore masks when going out (2,037, 90.7%). Many respondents kept their hands clean (1,881, 83.8%), did not go out (1,902, 84.7%), and ventilated their residences (1,843, 82.1%). The health behavior score of students was 15.05 ± 2.50 points (range: 0–20 points).

**Table 4:** Comparison of COVID-19 practice scores among medical students.

Variable categories	Score	Number of participants (%)	Score(*x̅* ± s) (point)	Score (*x̅* ± s) (point)
Disinfected the residence	2	1,641 (73.1)	1.72 ± 0.48	15.05 ± 2.50
	1	571 (25.4)		
	0	33 (1.5)		
Wash hands properly	2	1,881 (83.8)	1.83 ± 0.39	
	1	352 (15.7)		
	0	12 (0.5)		
Wear masks in public places	2	2,037 (90.7)	1.90 ± 0.32	
	1	195 (8.7)		
	0	13 (0.6)		
Ventilate the residence	2	1,843 (82.1)	1.81 ± 0.42	
	1	380 (16.9)		
	0	22 (1.0)		
Sleep time	2	668 (29.8)	1.25 ± 0.53	
	1	1,474 (65.6)		
	0	103 (4.6)		
Reasonable diet	2	462 (20.6)	1.14 ± 0.50	
	1	1,642 (73.1)		
	0	142 (6.3)		
Physical activity and exercise	2	321 (14.3)	0.68 ± 0.71	
	1	885 (39.4)		
	0	1,039 (46.3)		
Go out	2	1,902 (84.7)	1.26 ± 0.63	
	1	212 (9.4)		
	0	131 (5.9)		
Monitor body temperature	2	1,531 (68.2)	1.67 ± 0.49	
	1	690 (30.7)		
	0	24 (1.1)		
Study time	2	808 (36.0)	1.79 ± 0.53	
	1	1,202 (53.5)		
	0	235 (10.5)		

As shown in Table 5, the practice scores of female students were significantly higher than those of male students (*t* = 6.172, *P* < 0.001). Furthermore, significant differences were observed by household type, nation, and physical condition (*t* = 2.490, *t* = 2.257, and *t* = 9.487, respec*t*ively; *P* < 0.05).

**Table 5:** Comparison of health practice scores with different characteristics among medical students.

Variable categories		Score (x̅ ± s)	t	P
Sex	Male	14.38 ± 2.92	6.172	<0.001
	Female	15.25 ± 2.32		
Major	Clinical medicine	15.18 ± 2.45	1.07	0.370
	Preventive medicine	14.90 ± 2.33		
	Nursing	15.02 ± 2.58		
	Clinical pharmacy	15.00 ± 2.71		
	Health inspection and quarantine	15.03 ± 2.58		
Household type	One-child family	14.82 ± 2.47	2.490	0.013
	Non-one-child family	15.12 ± 2.50		
Grade	Freshman	15.23 ± 2.33	1.235	0.295
	Sophomore	14.95 ± 2.44		
	Junior	14.96 ± 2.64		
	Senior	15.01 ± 2.60		
	Fifth grade	15.12 ± 2.13		
Annual per capita household income (yuan)	<10000	15.04 ± 2.49	0.058	0.994
	10000–13999	15.07 ± 2.61		
	14000–16999	15.05 ± 2.48		
	17000–19999	15.15 ± 2.07		
	≥20000	15.05 ± 2.45		
Age	≤19	15.13 ± 2.50	1.933	0.145
	20–24	15.04 ± 2.50		
	≥25	13.94 ± 2.77		
Nation	Han	15.08 ± 2.48	2.257	0.026
	Non-Han	14.38 ± 2.88		
Physical condition	Healthy	15.14 ± 2.48	9.487	<0.001
	Fine	14.92 ± 2.46		
	Common	13.65 ± 2.55		
	Poor	14.40 ± 3.58		

## 4. Discussion

As a classic theory of behaviour change, the KAP model fundamentally aims to elucidate the progressive relationship between knowledge, attitudes, and practices, ultimately facilitating the establishment of positive health behaviours through effective interventions [24]. This theory posits that knowledge accumulation forms the foundation, correct beliefs and attitudes serve as the driving force, while ultimate behavioural change constitutes the objective. This framework not only serves as an effective tool for assessing individual health literacy but also constitutes the theoretical bedrock for designing and evaluating public health interventions.

The significance of this theory is particularly pronounced in the context of EIDs prevention and control. EIDs are characterised by high uncertainty, rapid transmission, and significant societal harm [25]. Their effective control relies heavily on healthcare workers—particularly medical students, who represent the future workforce—being able to promptly access accurate knowledge, develop scientifically grounded prevention beliefs, and translate these into correct reporting, protective, and diagnostic practices. Their level of knowledge, belief, and action directly impacts the speed and efficacy of the “first response” in future epidemic management.

Although the significance of the knowledge-attitude-behaviour theory is self-evident, systematic investigations into the current state of knowledge, attitudes, and behaviours regarding EIDs among medical students—a critical cohort—remain relatively scarce. This study found that respondents’ overall awareness of COVID-19 prevention knowledge reached 91.2%, exceeding that of other groups, potentially due to students’ greater exposure to medical knowledge [26]. Among them, 96.4% of participants (2,165 individuals) could accurately identify the primary transmission routes of COVID-19, a critical understanding that aids in avoiding infection. Regarding specific protective measures, 97.5% of respondents (n = 2189) recognised the importance of selecting appropriate masks, consistent with prior KAP studies [26]. Concurrently, 91.4% of participants (2051 individuals) understood the general susceptibility of populations to coronaviruses, underscoring the importance of proactive protective measures for safeguarding personal and communal health. Furthermore, the study observed heightened public environmental awareness, with 87.5% of respondents (1,965 individuals) knowing the correct disposal method for used masks. Regarding personal hygiene practices, 78.4% of participants (1,759 individuals) understood the proper conduct when sneezing or coughing, consistent with prior research findings [27], indicating that most have adopted hygiene habits that reduce disease transmission risks. Notably, 95.0% of respondents (2,133 individuals) clearly understood the required isolation period following close contact, reflecting a relatively comprehensive public understanding of epidemic prevention measures.

During the pandemic, the public gained a basic understanding of viral transmission mechanisms and protective measures. However, there remained insufficient awareness regarding the pathogen characteristics of emerging infectious diseases and long-term prevention strategies. This cognitive limitation was particularly pronounced during the early stages of the outbreak and could potentially trigger panic. The survey revealed that 96% of participants (2,153 individuals) believed COVID-19 had severely disrupted their normal lives, altering work patterns and social habits while imposing psychological and economic pressures. This impact varied across age groups, diminishing with increasing age. Additionally, 32.6% of students (731 individuals) exhibited negative attitudes towards the local epidemic situation. This may stem from risk assessment biases caused by information asymmetry and delayed scientific understanding, leading to neglect of protective measures. The findings align with the Knowledge-Attitude-Practice (KAP) model, wherein knowledge levels constitute a key determinant of health behaviours [28]. Future public health education should strengthen science communication regarding novel pathogens, particularly zoonotic risks, and enhance public preparedness through scenario-based simulations.

The survey revealed that 83.8% of students maintained good hand hygiene and 90.7% consistently wore masks, indicating that most students implemented key protective measures [29]. However, 5.9% of students still frequently go out, and 46.3% have significantly reduced their physical activity levels. These findings suggest that some students have weak epidemic prevention awareness and that EIDs may impact their physical and mental health. This strongly indicates that current infectious disease curricula may still focus predominantly on classical infectious diseases, with insufficient coverage of the latest epidemiological characteristics of emerging infectious diseases. Concurrently, schools lack adequate attention and effective guidance regarding students’ mental health. Therefore, in combating emerging infectious diseases, the foremost task is to systematically reconstruct teaching content based on these knowledge gaps, strengthen health education, and integrate routine health management (such as indoor exercise) into emergency response systems. This will foster students’ rational understanding of epidemic prevention and promote stable psychological states.

Research indicates that effectively addressing emerging infectious diseases requires three key elements. Firstly, individuals must possess accurate scientific knowledge about disease characteristics and the ability to identify misinformation. Secondly, individuals must maintain a rational attitude towards epidemics while upholding a spirit of mutual support. Finally, this understanding must translate into concrete actions—individuals need to practise correct prevention methods, counter misinformation, and fulfil social responsibilities. Research indicates that integrating knowledge, attitudes, and practical measures yields the most effective public health responses.

This study has certain limitations. Firstly, the questionnaire employed in the research has not undergone systematic validation, which may impact the accuracy and generalisability of the measurement results. Secondly, the findings may exhibit discrepancies relative to societal expectations. Furthermore, the cross-sectional study design precludes the establishment of causal relationships between variables.

In summary, the majority of students at Medical University demonstrated positive attitudes and low-risk practices regarding infectious diseases. However, a minority exhibited insufficient knowledge, while some displayed negative attitudes or high-risk behaviours.

## Supporting information

S1 File The study design and reporting strictly followed the Enhancing the QUAlity and Transparency Of health Research (EQUATOR) guidelines, with cross-sectional components adhering to the Strengthening the Reporting of Observational Studies in Epidemiology (STROBE) statement, including its checklist. This section is reflected in the study procedures and data collections, and the checklist can be found in the appendix.
(DOCX)
